# Mammographic Findings of Diffuse Axillary Tail Trabecular Thickening After the Second Booster of COVID-19 Vaccination

**DOI:** 10.7759/cureus.29993

**Published:** 2022-10-06

**Authors:** Richard Adam, Laura Hodges, Tim Q Duong, Takouhie Maldjian

**Affiliations:** 1 Radiology, Montefiore Medical Center and Albert Einstein College of Medicine, Bronx, USA

**Keywords:** axillary tail, mammography, trabecular thickening, covid-19 retro, covid-19 vaccine booster shot

## Abstract

Axillary adenopathy post-coronavirus disease 2019 (COVID-19) vaccination has been well-documented and is seen with other types of vaccinations. Isolated trabecular thickening on mammography, however, is singular to COVID-19 vaccination, which implies that this finding may result from a distinct pathophysiologic mechanism. Herein, we describe the first case of axillary tail trabecular thickening resulting from the second booster of the COVID-19 vaccination series. Both breast cancer and mastitis may present similar findings. Ipsilateral injection of COVID-19 vaccine/booster and spontaneous resolution on follow-up provide clues to the etiology. It has been hypothesized that proinflammatory conditions may predispose to axillary tail trabecular thickening on mammography post-COVID-19 vaccination. Proinflammatory conditions such as hypertension, obesity, and diabetes may also predispose to breast cancer, making this scenario even more of a diagnostic dilemma. This scenario would more likely be seen in lower socioeconomic communities, African Americans, and Hispanics, who demonstrate a higher prevalence of these diseases, and who are also more vulnerable due to health care disparities negatively affecting these groups. We discuss our case and the importance of this public health issue. Sequela of COVID vaccination and boosters will be encountered in the foreseeable future and could pose a diagnostic dilemma, thus potentially straining the healthcare system with unnecessary biopsies and patient anxiety if not recognized and appropriately managed.

## Introduction

In this case report, we describe the mammographic finding of isolated trabecular thickening in an asymptomatic patient presenting for screening mammography shortly after the second booster of the coronavirus disease 2019 (COVID-19) vaccine in the ipsilateral arm. This is the first case report in a patient after a second booster. COVID-19 vaccination and periodic boosters are an integral part of the continual global effort against the COVID-19 pandemic. Sequela of COVID-19 vaccination and boosters will therefore be encountered in the foreseeable future and could pose a diagnostic dilemma, thus potentially straining the healthcare system with unnecessary biopsies and patient anxiety.

## Case presentation

**Figure 1:**
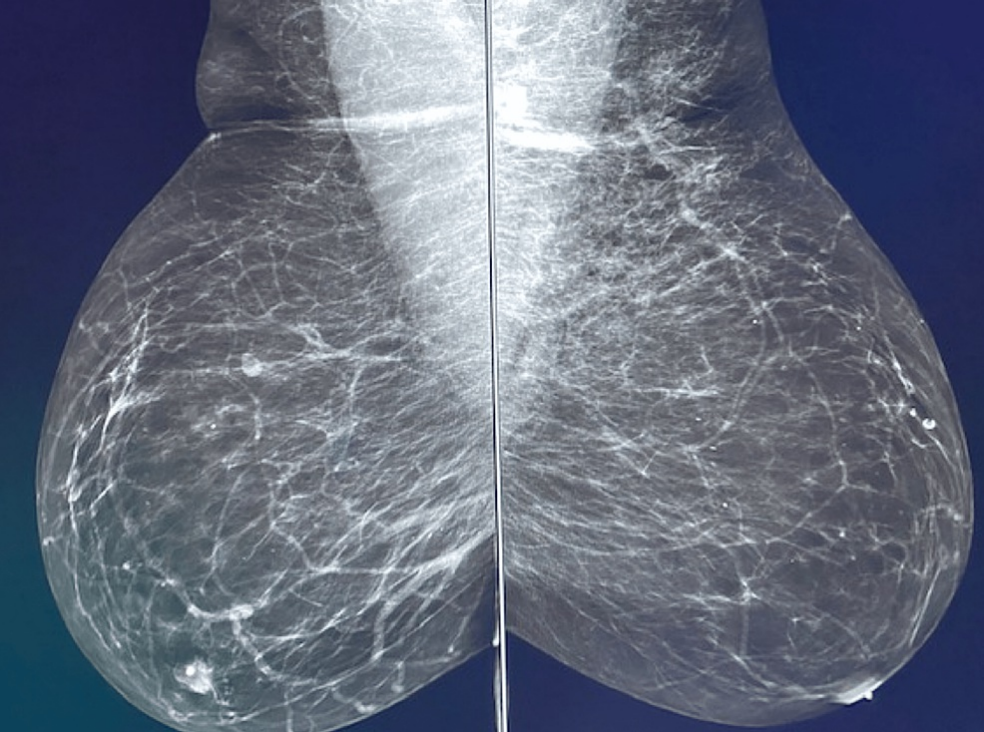
Bilateral mediolateral oblique (MLO) view from screening mammogram. The mammogram demonstrates trabecular thickening of the left axillary tail.

An asymptomatic patient presented for screening mammography four days after receiving her second Pfizer booster COVID-19 vaccination in the left arm. A screening mammogram demonstrated diffuse axillary tail trabecular thickening of the left breast (Figure 1). The mammogram was interpreted as BI-RADS 0 and evaluation with spot compression was recommended.

**Figure 2:**
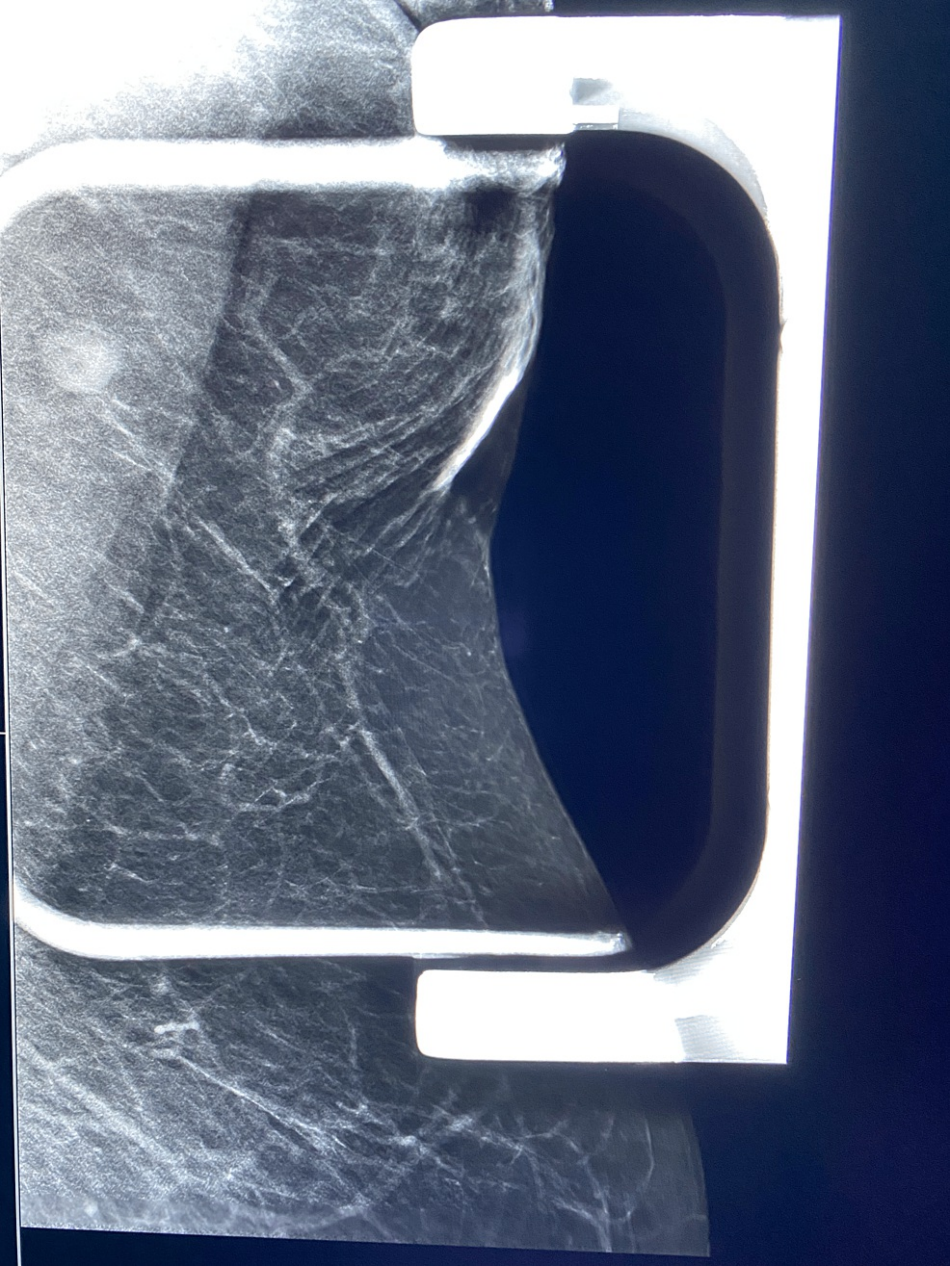
Spot compression left MLO view obtained with 3D technique at follow-up diagnostic mammogram four weeks later MLO: Mediolateral oblique The mammogram demonstrates spontaneous resolution of the trabecular thickening.

Diagnostic mammography with spot compression 3-D imaging performed about four weeks later demonstrated the resolution of axillary tail trabecular thickening (Figure 2). Ultrasound was also performed as part of the diagnostic examination and demonstrated a single normal axillary lymph node and no abnormality was seen. Pertinent medical history included hypertension, hyperlipidemia, diabetes, and morbid obesity. Antihypertensive medications that the patient is taking include nifedipine, furosemide, and spironolactone. The patient's hemoglobin (Hb)A1c level is above 6.5%, and the patient's diabetes is controlled by diet without the use of medication. The patient had a history of elevated triglycerides which is currently under control.

## Discussion

To our knowledge, this is the first case of isolated trabecular thickening on screening mammography as a result of the second booster, after completion of a primary COVID-19 vaccination series and first booster, demonstrating that such findings will likely continue to be seen with continued boosters.

The first and largest case series of ipsilateral changes of diffuse axillary tail trabecular thickening at screening mammography as an isolated finding within one week of COVID-19 vaccination or first booster was recently reported [1]. Five patients from a mammography screening population were reported as having reversible axillary tail trabecular thickening with or without axillary adenopathy in connection with the first COVID-19 vaccination or first booster [1]. This trabecular thickening likely represents tissue edema. Four of the five patients had hypertension and/or obesity which was hypothesized may predispose to these findings through an immune-mediated mechanism heightened by underlying pro-inflammatory conditions [1]. The three patients with isolated trabecular thickening were observed to have underlying conditions of hypertension and obesity; hypertension obesity, and scleroderma; and obesity and prediabetes [1]. This was the first publication linking the changes of axillary tail trabecular thickening with or without ipsilateral adenopathy to comorbidities known to be associated with the severity of outcomes of COVID-19 infection [1-3]. This suggests that populations with underlying proinflammatory conditions and comorbidities are at greater risk for developing these findings which typically occur within the first week after vaccination. Additionally, post-vaccination changes in the breast may potentially obfuscate other underlying pathology, further complicating the issue since the post-vaccination changes are typically seen in patients with comorbidities that also place one at higher risk for breast cancer [4].

It is intriguing that while other types of vaccinations have been shown to produce axillary lymphadenopathy, the changes intrinsic to the breast appear to be unique to COVID-19 vaccination [5-7]. Severe acute respiratory syndrome coronavirus 2 (SARS-CoV-2) antibodies react with 28 of 55 human tissue antigens [8]. The mRNA vaccine results in the synthesis of a portion of the spike protein, which stimulates the immune system to synthesize antibodies. Cross-reactivity of these antibodies to human tissue explains myocarditis post-vaccination [9,10]. It has been thus hypothesized that similar cross-reactivity with human tissues could explain the temporary trabecular thickening in the axillary tail [1]. Based on this hypothesis, this finding is generated through an immune-mediated mechanism heightened by underlying pro-inflammatory conditions [1]. The connection with proinflammatory conditions, such as diabetes, obesity, hypertension, and hyperlipidemia which were all present in our patient, therefore makes sense from a pathophysiologic standpoint. Given that comorbidities often seen with axillary tail trabecular thickening also predispose to breast cancer, it is important to obtain follow-up imaging. Follow-up diagnostic imaging to document resolution has been advocated to exclude other diagnoses [1]. Follow-up imaging showed complete resolution of the findings in our case, confirming that it was the sequela of the second COVID-19 vaccine booster.

## Conclusions

We present findings of axillary tail trabecular thickening on screening mammography in an asymptomatic patient post-second COVID-19 vaccine booster. This is the first such case reported in connection with the second booster, indicating that these findings will likely continue to be seen, as COVID-19 vaccination/boosters will continue to be administered, requiring appropriate management. Proinflammatory conditions such as hypertension, obesity, and diabetes may predispose to breast cancer as well as post-COVID-19 vaccination trabecular thickening, and to complicate this further, post-COVID-19 vaccine trabecular thickening may obfuscate a concomitant underlying cancer, making this scenario even more of a diagnostic dilemma and challenge. Post-COVID-19 vaccine trabecular thickening would more likely be seen in lower socioeconomic communities, African Americans, and Hispanics, due to the higher prevalence of the predisposing comorbidities (hypertension, obesity, and diabetes) in these groups that are already more vulnerable due to adverse effects of health disparities and health inequities. These factors further highlight the importance of this public health issue.

The main differential diagnosis for unilateral axillary tail trabecular thickening is mastitis and inflammatory breast cancer, however, skin thickening often seen with these diagnoses was not observed in our case. Including ultrasound in the diagnostic work-up may be beneficial in arriving at the proper diagnosis. It is important to recognize these findings in the context of COVID-19 vaccinations and boosters to avoid unnecessary biopsies that would strain the healthcare system and to avoid needless patient anxiety.
